# Epileptic seizure prediction based on EEG using pseudo-three-dimensional CNN

**DOI:** 10.3389/fninf.2024.1354436

**Published:** 2024-03-19

**Authors:** Xin Liu, Chunyang Li, Xicheng Lou, Haohuan Kong, Xinwei Li, Zhangyong Li, Lisha Zhong

**Affiliations:** ^1^Research Center of Biomedical Engineering, Chongqing University of Posts and Telecommunications, Chongqing, China; ^2^School of Automation, Chongqing University of Posts and Telecommunications, Chongqing, China; ^3^School of Communication and Information Engineering, Chongqing University of Posts and Telecommunications, Chongqing, China; ^4^School of Bioinformatics, Chongqing University of Posts and Telecommunications, Chongqing, China; ^5^School of Medical Information and Engineering, Southwest Medical University Luzhou, Luzhou, China

**Keywords:** epilepsy, feature selection, MRMR, pseudo-3D CNN, seizure prediction

## Abstract

Epileptic seizures are characterized by their sudden and unpredictable nature, posing significant risks to a patient’s daily life. Accurate and reliable seizure prediction systems can provide alerts before a seizure occurs, as well as give the patient and caregivers provider enough time to take appropriate measure. This study presents an effective seizure prediction method based on deep learning that combine with handcrafted features. The handcrafted features were selected by Max-Relevance and Min-Redundancy (mRMR) to obtain the optimal set of features. To extract the epileptic features from the fused multidimensional structure, we designed a P3D-BiConvLstm3D model, which is a combination of pseudo-3D convolutional neural network (P3DCNN) and bidirectional convolutional long short-term memory 3D (BiConvLstm3D). We also converted EEG signals into a multidimensional structure that fused spatial, manual features, and temporal information. The multidimensional structure is then fed into a P3DCNN to extract spatial and manual features and feature-to-feature dependencies, followed by a BiConvLstm3D input to explore temporal dependencies while preserving the spatial features, and finally, a channel attention mechanism is implemented to emphasize the more representative information in the multichannel output. The proposed has an average accuracy of 98.13%, an average sensitivity of 98.03%, an average precision of 98.30% and an average specificity of 98.23% for the CHB-MIT scalp EEG database. A comparison of the proposed model with other baseline methods was done to confirm the better performance of features through time–space nonlinear feature fusion. The results show that the proposed P3DCNN-BiConvLstm3D-Attention3D method for epilepsy prediction by time–space nonlinear feature fusion is effective.

## 1 Introduction

Epilepsy is a neurological disorder characterized by epileptic seizures (Chang and Lowenstein, 2003; Fisher et al., 2014), which are often accompanied by intense shaking or convulsions. According to statistics from the World Health Organization, neurological disorders rank as the second leading cause of global mortality. It is estimated that an additional 5 million individuals are diagnosed with epilepsy worldwide annually (World Health Organization, 2022). Therefore, epilepsy deserves significant attention and focus to improve its prevention and treatment efforts. The unpredictability, suddenness, and recurrence of epileptic seizures can cause additional anxiety for individuals with epilepsy and their families. Epilepsy also has a negative impact on society, as the stigma and bias against individuals with epilepsy can lead to feelings of shame and social isolation for the affected individuals. This stigma can hinder societal development and progress. Therefore, epilepsy prediction and treatment became particularly important. Seizures are controllable with medication in about 70% of cases, so early prediction of epilepsy reduces the worry about epilepsy, as having enough period to stop a seizure before it occurs reduces the patient’s suffering to a great extent.

Seizure prediction is one of the hot topics in clinical research, which is a challenging task. Seizures are the result of excessive and abnormal neuronal activity in the cerebral cortex, so epilepsy can usually be detected by electroencephalography (EEG). EEG reflects the electrical activity of neurons in the brain, and more than 80% of people with epilepsy can be monitored for abnormalities by EEG. Therefore, it is of great value to analyze EEG in the diagnosis of epilepsy. With the development of modern science, a variety of methods have been developed to automatically predict seizures. Most of these methods are based on EEG analysis.

In the literature, there are several prediction methods that can be used to confirm the challenge of predicting seizures. The combination of manual feature extraction from time-series signals and traditional machine learning classifiers has indeed made significant contribution to epilepsy detection (Sharma et al., 2019). Lu et al. (2021) employed support vector machines for automatic classification of epileptic EEG signals. They chose sample entropy and Higuchi fractal dimension as features, and achieved 89.8% accuracy. Non-linear features show effectiveness in epilepsy detection or prediction. Manual feature makes the model easier to interpret and better able to capture the essential features of the data. It can also be customized for different research tasks and applications. Our purpose is to explore the prediction of epilepsy based on nonlinear features. Appropriate feature selection determines the accuracy of the system, but relying only on features and SVM cannot adequately access the hidden information of the data, requiring a combination of other techniques and methods. With the development of neural networks, various neural network methods are gradually being applied to the detection and prediction of epilepsy. Among these neural networks, recurrent neural networks, convolutional neural networks and graphical neural networks have become prominent. He et al. (2022) utilized a graph attention network as the front end to extract spatial features, and used a bidirectional long short-term memory network as the back end to capture temporal relationships. As a result, the seizure detection accuracy on CHB-MIT is 98.52%. Yu et al. (2022) utilized manual features and hidden deep features for complementary fusion through the feature fusion module. These fused features were then input into a Multiplicative Long Short-Term Memory network, achieving an average sensitivity of 95.56% and a false positive rate of 0.27/h. In addition, neural networks have been proved to be effective in epilepsy detection or prediction. we will further study neural network epilepsy prediction. Singh and Malhotra (2022) using the spectral power and average spectral amplitude of each band as the characteristic inputs of the two-layer LSTM, and achieved 98.14% accuracy, 98.51% sensitivity and 97.78% specificity. Zhang et al. (2021) combined with multidimensional sample entropy and Bi-LSTM, the seizure prediction accuracy was 80.09% and the FPR was 0.26/h. Tuncer and Bolat (2022) using EEG instantaneous frequency and spectral entropy as features, Bi-LSTM can also be used to classify seizures well. The results show that the combination of artificial features and Bi-LSTM still has high efficiency in predicting seizures. Prathaban and Balasubramanian (2021) reconstructed the EEG with sparse and converted it into a two-dimensional image. Then, in order to explain the relationship between channels, the 2D image is transformed into a three-dimensional image of time, signal value and channel representation, and a 3D optimized convolutional neural network was used to predict epileptic seizures. It shows that epilepsy prediction based on the 3D neural network can be realized. However, it should be noted that features with high redundancy can affect the performance of the model. Only by selecting features and reducing redundancy between features can we improve the calculation efficiency of the model and optimize the performance of the model. Xing et al. (2022) segmented EEG signals into five frequency bands: α, β, γ, θ, and δ, calculated their power spectral density values, merged spatial information from multiple electrodes, and then applied them to a 3D neural network, a bidirectional long and short-term memory network. This method successfully realized the emotion classification. The study also incorporated spatial information from electrodes into the analysis of emotion recognition. The principle of EEG acquisition is the waveform of the potential difference between two electrodes on the scalp, so the position of the electrodes reflects the state of other adjacent electrodes. This means that we can get some information about the EEG signal from the spatial information of the electrodes. Therefore, we will select features, combines manual features, 3D neural network, and Bi-ConvLSTM3D to form a neural network structure model that preserves spatial information: P3DCNN-BiConvLSTM3D-Attention3D. Using this model can better intervene epileptic seizures and reduce the negative impact of epilepsy.

The article is structured as follows: In Section 2, we provide a brief description of the dataset, signal pre-processing, selected feature types, mRMR algorithm, and 3D feature construction. Section 3 presents an overview of the EEG spatial information modelling, P3DCNN-BiConvLSTM3D-Attention3D model application, and evaluation metrics. We then discuss and compare the results with previous studies. Finally, we provide our conclusions.

## 2 Materials and methods

Epileptic signals are essentially nonlinear, so nonlinear characteristics are part of the research. Using a single feature may not be able to effectively capture epilepsy-related information, and too many features will reduce the efficiency of the algorithm. Therefore, multiple features are used to represent the features of epileptic signals. The Max-Relevance and Min-Redundancy algorithm (mRMR) is used to select important non-linear features while maximizing their relevance and minimizing redundancy.

**Figure 1:**
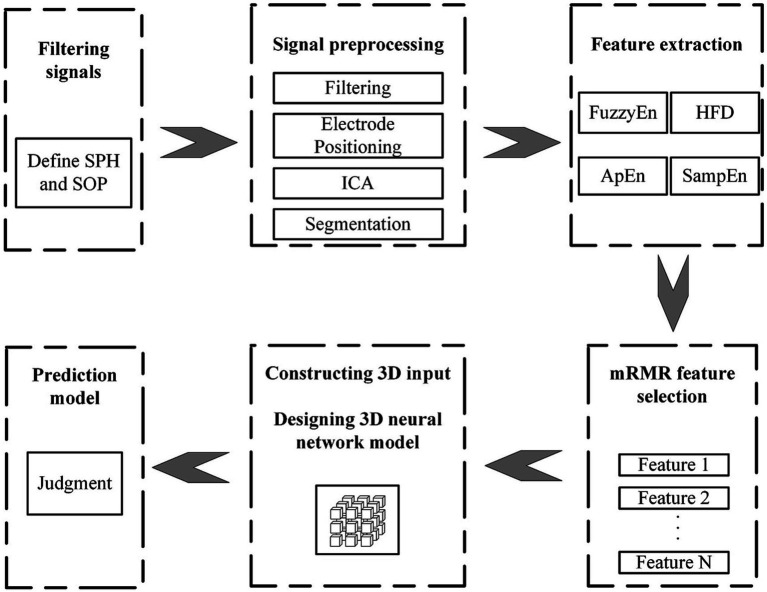
Flow chart of multi feature selection and temporal spatial epilepsy prediction.

Figure 1 shows the algorithmic process of this study. The process begins with the selecting and preprocessing of EEG signals from the dataset. Following this, several feature are extracted. Apply the mRMR algorithm to obtain highly significant features and then combine them with the spatial relationship of the electrode channels to create 3D features with spatial features. Input 3D features into the P3DCNN Biconvlstm3D model, and finally add the channel attention mechanism to improve the performance and efficiency of the model. The KNN and SVM are used to conduct synchronous comparison experiments.

### 2.1 CHB-MIT dataset

The present work used a public dataset created jointly by Children’s Hospital Boston (CHB) and the Massachusetts Institute of Technology (MIT). The dataset is called CHB-MIT. The dataset contains 24 cases, of which 21 cases and 1 cases were from the same female patient, and an interval between data collection was one and a half years. The participants included 5 males ranging in age from 3 to 22 and 17 females ranging in age from 1.5 to 19. The dataset consists of 967.55 h of scalp EEG records, including 178 recorded seizures.

### 2.2 Preprocessing of EEG signals

Due to the low amplitude of EEG signals, they are susceptible to external environmental interference, such as powerline frequency (60 Hz or 50 Hz) interference. In addition, physiological activity can introduce artifacts into EEG signals, mainly including eye artifacts and muscle artifacts caused by eye movement and blinking. Therefore, in order to obtain relatively clean EEG signals, signal preprocessing must be done before feature extraction. Firstly, the EEG signals is filtered by using a band-pass filter in the range of 0.5 to 75 Hz. Because this study needed to consider the influence of electrode placement, it is very important to locate the electrodes in the preprocessing phase. Use the pop_chanedit function in EEGLAB to locate scalp electrode. The EEG signals were processed using EEGLAB’s Independent Component Analysis (ICA) through the pop_runica function. Use pop_selectcomps function to remove these components manually to obtain relatively clean EEG. The processed EEG signals were subsequently segmented.

### 2.3 Feature type selection

Nonlinear dynamics analysis methods may better suit for analysis of the complex and nonlinear EEG waveform recorded from the brain than traditional linear methods, such as time and frequency domain analysis. Nonlinear features can effectively capture the characteristics of biological systems, and can also be used in the analysis of EEG (Acharya et al., 2013). Due to the instability and non-stationary of epileptic signals, we extract the following non-linear features from the EEG signals: Higuchi Fractal Dimension (HFD) (Sharma and Joshi, 2022), Approximate Entropy (ApEn) (Srinivasan et al., 2007), Sample Entropy (SampEn) (Arunkumar et al., 2016), and Fuzzy Entropy (FuzzyEn) (Xiang et al., 2015), FuzzyEn works equally well for fuzzy time series and can describe the degree of ambiguity of the series (Versaci and Morabito, 2003). These nonlinear features were subjected to feature selection.

Fractal dimension is a measure used to quantify the complexity of signals. In this study, we used HFD to characterize the fractal dimension of the signal. HFD is computed by the following steps (Higuchi, 1988):

Step 1: constructing a new time series as Eq. (1):


(1) $$x_{m}^{k}=\left\{x\left(m\right),x\left(m+k\right),x\left(m+2k\right),\ldots ,x\left(m+\left[\frac{N-m}{k}\right]\times k\right)\right\}$$

Step 2: Eqs. (2) and (3)can be used to calculate the duration of the time series.


(2) $$L_{m}\left(k\right)=\frac{1}{k}\left(\overset{\left[\frac{N-m}{k}\right]}{\underset{i=1}{\sum }}\left|x\left(m+ik\right)-x\left(m+\left(i-1\right)k\right)\right|\right)\frac{N-1}{\left[\frac{N-m}{k}\right]\cdot k}$$

(3) $$L\left(k\right)=\frac{1}{k}\overset{k}{\underset{m=1}{\sum }}L_{m}\left(k\right)$$

Step 3: the HFD is calculated as follows Eq. (4):


(4) $$D=\frac{\ln\left(L\left(k\right)\right)}{-\ln\left(k\right)}$$

ApEn is an index to measure the complexity of time series. It is a nonlinear dynamics parameter, which is used to measure regularity and volatility of time series by comparing the similarity of template vectors. ApEn is computed as below (Pincus, 1991):

In general, for a time series $x\left(n\right)=x\left(1\right),x\left(2\right),..,x\left(N\right)$ consisting of N data points, the method for calculating ApEn is as follows:

First, constructing an m-dimensional vector $X_{1}^{m},\ldots ,X_{N-m+1}^{m}$ , where $X_{i}^{m}=\left\{x\left(i\right),x\left(i+1\right),\ldots ,x\left(i+m-1\right)\right\},1\leq i\leq N-m+1$ .

Second, define the distance $d_{ij}^{m}$ between vectors $X_{i}^{m}$ and $X_{j}^{m}$ as the Chebyshev distance as Eq. (5) which is the maximum absolute difference between their corresponding elements.


(5) $$d_{ij}^{m}=D_{\mathrm{chebychev}}\left(X_{i}^{m},X_{j}^{m}\right)=\max_{k=0,\ldots ,m-1}|x\left(i+k\right)-x\left(j+k\right)|$$

Third, count the number of j for which $d_{ij}^{m}$ is less than or equal to the similarity threshold r , and define the approximate count $c_{i}$ . For 1≤i≤N−m+1 , $c_{i}^{m,r}$ is designated as the ratio of the approximate count to the total count as Eq. (6).


(6) $$c_{i}^{m,r}=\frac{1}{N-m+1}c_{i}$$

Fourth, define $\phi^{m,r}$ as Eq. (7):


(7) $$\phi^{m,r}=\frac{1}{N-m+1}\overset{N-m+1}{\underset{i=1}{\sum }}\ln c_{i}^{m,r}$$

Fifth, increase the dimension to m+1 and obtain $\phi^{m+1,r}$ .

Sixth, define ApEn as Eq. (8):


(8) $$\mathrm{ApEn}\left(m,r\right)=\phi^{m,r}-\phi^{m+1,r}$$

SampEn is an improvement on ApEn. The following steps are used to calculate SampEn (Richman and Moorman, 2000):

For a sequence $x\left(n\right)$ , calculate the maximum distance between $X\left(i\right)$ and $X\left(j\right)$ as $d\left[X\left(i\right),X\left(j\right)\right]=\max_{k\in \left(0,m-1\right)}\left|x\left(i+k\right)-x\left(j+k\right)\right|$ . Then calculate the ratio relationship: $B_{i}^{m}\left(r\right)=\frac{1}{N-m}num\left\{d\left[X\left(i\right),X\left(j\right)\right]<r\right\}$ , where r is the similarity threshold. $B^{m}=\frac{1}{\left(N-m+1\right)\sum_{i=1}^{N-m+1}B_{i}^{m}\left(r\right)}$ . Next, increase the dimension to m+1 and obtain $B^{m+1}\left(r\right)$ . The formula for calculating SampEn is as Eq. (9):


(9) $$\mathrm{SampEn}\left(m,r,N\right)=-\ln\left[\frac{B^{m+1}\left(r\right)}{B^{m}\left(r\right)}\right]$$

$B^{m}$ is never equal to zero. This is because the distance between each pair of vectors $X\left(i\right)$ and $X\left(j\right)$ is greater than zero, and the value of $B_{i}^{m}\left(r\right)$ is always greater than zero. So the value of $B^{m}$ is always greater than zero.

FuzzyEn is used to measure the uncertainty or information content of fuzzy sets or fuzzy systems. FuzzyEn is defined as Chen et al. (2007):

To calculate the mean-removed template vector, $X\left[i\right]=\left[x\left[i\right]x\left[i+1\right]\ldots x{\left[i+m-1\right]}^{T}-\bar{x}\left[i\right]\right]\text{,}$ where $\bar{x}\left[i\right]=\left(1/m\right)\overset{m-1}{\underset{j=0}{\sum }}x\left[i+j\right]$ . The Gaussian function definition is employed Eqs. (10–12):


(10) $$\Phi^{m}\left(r\right)=\frac{1}{N-m+1}\overset{N-m+1}{\underset{i=1}{\sum }}\left(\frac{1}{N-m}\overset{N-m+1}{\underset{j=1,j\neq i}{\sum }}D_{i,j}^{m}\right)$$

(11) $$D_{i,j}^{m}=\exp\left[-\frac{{\left(d_{i,j}^{m}\right)}^{n}}{r}\right]$$

(12) $$d_{i,j}^{m}=d\left[X\left[i\right],X\left[j\right]\right]=\max_{k\in \left(0,m-1\right)}|\begin{array}{l} \left(x\left(i+k\right)-\bar{x}\left[i\right]\right)\\ -\left(x\left(j+k\right)-\bar{x}\left[j\right]\right) \end{array}|$$

The formula for computing FuzzyEn is as follows Eq. (13):


(13) $$\mathrm{FuzzyEn}\left(X,m,r\right)=\log\Phi^{m}\left(r\right)-\log\Phi^{m+1}\left(r\right)$$

### 2.4 Feature filtering based on mRMR

This algorithm is to find a set of features in the original feature set that have the max-relevance with the final output result, but have the min-redundancy between the features. In order to minimize the redundancy of features and obtain the most information with the least features, we use the mRMR method to select feature (Peng et al., 2005). Using this algorithm, we can choose the features with the highest information, thus improving the performance and accuracy of the model. The experimental process is as follows: we subject pre-processed EEG signals to feature extraction and use the mRMR method to select feature group with the max-relevance and min-redundancy. Effective feature selection can extract highly correlated features for epilepsy detection, while eliminating those features with poor correlation. The combination of these features better captures the integrity of the signal, reduces the complexity and improving the efficiency of network learning. This method achieves the highest accuracy with fewer features, mRMR is computed by the following steps (Peng et al., 2005):

Define the mutual information between $x_{i}$ and $x_{j}$ as Eq. (14):


(14) $$I\left(x_{i};x_{j}\right)=\iint p\left(x_{i},x_{j}\right)\log\frac{p\left(x_{i},x_{j}\right)}{p\left(x_{i}\right)p\left(x_{j}\right)}dx_{i}dx_{j}$$

Using mutual information, the mRMR criterion can be obtained as Eqs. (15, 16):


(15) $$\max D\left(S,c\right),D=\frac{1}{\left|S\right|}\underset{x_{i}\in S}{\sum }I\left(x_{i};c\right)$$

(16) $$\min R\left(S\right),R=\frac{1}{{\left|S\right|}^{2}}\underset{x_{i},x_{j}\in S}{\sum }I\left(x_{i};x_{j}\right)$$

Where S represents the feature set, with |S| being the dimensionality. $I\left(x_{i};c\right)$ represents for the mutual information between feature $x_{i}$ and target c , while $I\left(x_{i};x_{j}\right)$ represents for the mutual information between $x_{i}$ and $x_{j}$ . D and R denote the relevance and redundancy, respectively.

The mRMR algorithm considers both of the above criteria as Eq. (17):


(17) $$\max\Phi \left(D,R\right),\Phi =D-R$$

To solve the equation above, we use an incremental search algorithm. That is, on the basis of the features that have been selected, find the one that maximises the Eq. (18) in the remaining feature space. In fact, it is equivalent to computing and then sorting each of the remaining features.


(18) $$\max_{x_{j}\in X-S_{m-1}}\left[I\left(x_{j};c\right)-\frac{1}{m-1}\underset{x_{i}\in S_{m-1}}{\sum }I\left(x_{i};x_{j}\right)\right]$$

**Figure 2:**
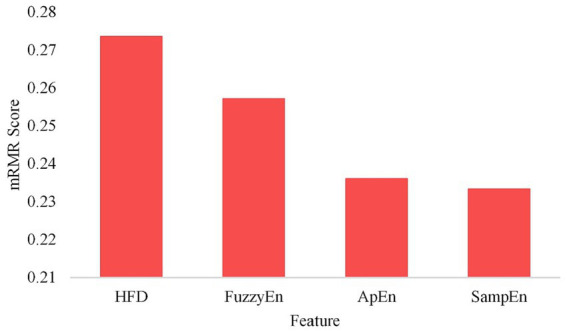
Importance score of each feature.

In the process of feature selection, the mRMR algorithm calculates feature importance based on HFD, ApEn, SampEn and fuzzy, and selects the most important variables one by one. Figure 2 shows the feature importance scores obtained by using the mRMR algorithm.

According to the results in Figure 2, it is obvious that this group of features are arranged in descending order of importance score: HFD, FuzzyEn, ApEn, and SampEn. It should be noted that SampEn has the lowest score, indicating that it has higher redundancy and relatively low correlation with other characteristics.

**Figure 3:**
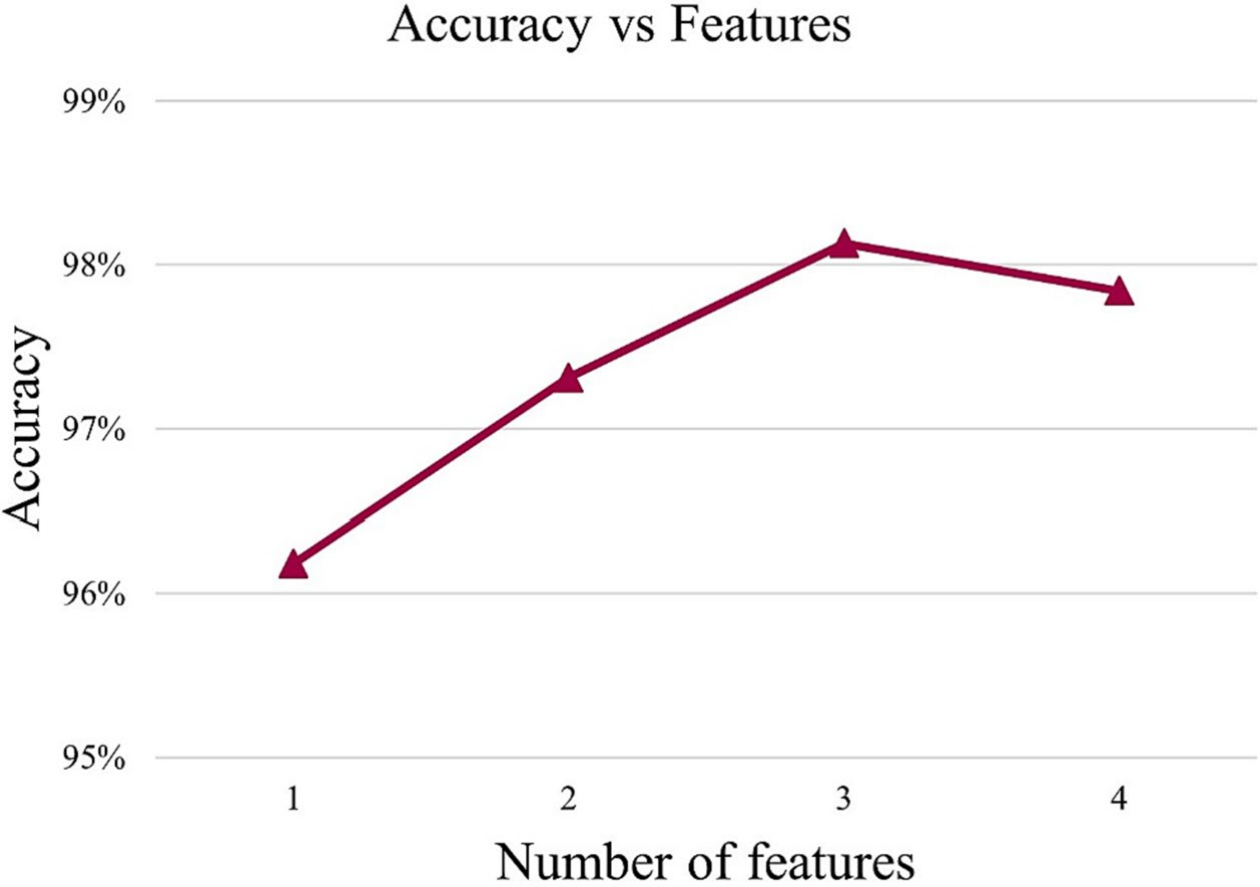
Accuracy values under different features.

In order to find the appropriate number of features, this article conducted experiments using different numbers of feature sets from high to low importance scores in HFD, FuzzyEn, ApEn, and SampEn. By comparing the effects of different feature numbers on the accuracy of the model, the optimal feature number is determined, as shown in Figure 3. It can be observed that with the increase of the number of features, the accuracy of the model is also improves, reaching the highest point at three features. However, with the addition of the fourth feature, the accuracy of the model drops. Therefore, it is very important to strike a balance between minimizing redundancy and maximizing relevance in the process of feature selection to ensure the best prediction performance. The effect of Figure 3 also reflects the correctness of the results of Figure 2. The model chosen is also the P3DCNN-BiConvLSTM3D-Attention3D model proposed in present work, which is selected using accuracy as an evaluation metric. The parameter settings are shown in Tables 1, 2.

**Table 1:** Pseudo 3D convolution feature extraction architecture.

Layer	Kernel size	Output size
P3DConv1	1 × 3 × 3 × 64	3 × 4 × 7 × 1
P3DConv2	3 × 1 × 1 × 64	3 × 4 × 7 × 64
P3DConv3	1 × 3 × 3 × 128	3 × 4 × 7 × 128
P3DConv4	3 × 1 × 1 × 128	3 × 4 × 7 × 128
P3DConv5	1 × 3 × 3 × 256	3 × 4 × 7 × 256
P3DConv6	3 × 1 × 1 × 256	3 × 4 × 7 × 256

**Table 2:** 3D RNN feature extraction architecture.

Layer	Size of hidden state	Output size
Input sequence	–	2 × 3 × 4 × 7 × 512
Bi-ConvLSTM3D layer	3 × 1 × 1 × 64	3 × 4 × 7 × 64
Fully connected layer	1 × 3 × 3 × 128	3 × 4 × 7 × 128

In conclusion, HFD, FuzzyEn and ApEn have been chosen as the features used in the experiments. The selected multiple features were combined with the 2D electrode channel spatial feature matrix. To normalize the data, import the StandardScaler class from the scikit-learn library and use the fit_transform method.

## 3 Epileptic-states classification

According to the EEG records of epileptic patients, their condition can be divided into two periods: the interictal period and the Seizure Occurrence Period (SOP). The interictal period represents to the time when the patient is in a normal state, and the SOP represents to the time range when the patient has epileptic symptoms. The main goal of epilepsy prediction is to detect seizures within the range of Seizure Prediction Horizon (SPH). An appropriate SPH should include an appropriate time range for taking adequate intervention or preventive measures before actual seizures. A long prediction range can cause patient anxiety and pose a challenge to using neural network prediction models, while a short prediction range may result in insufficient preparation time for patients and healthcare providers, ultimately failing to achieve the goal of epilepsy prediction. Achieving an appropriate balance is crucial in epilepsy prediction.

**Figure 4:**
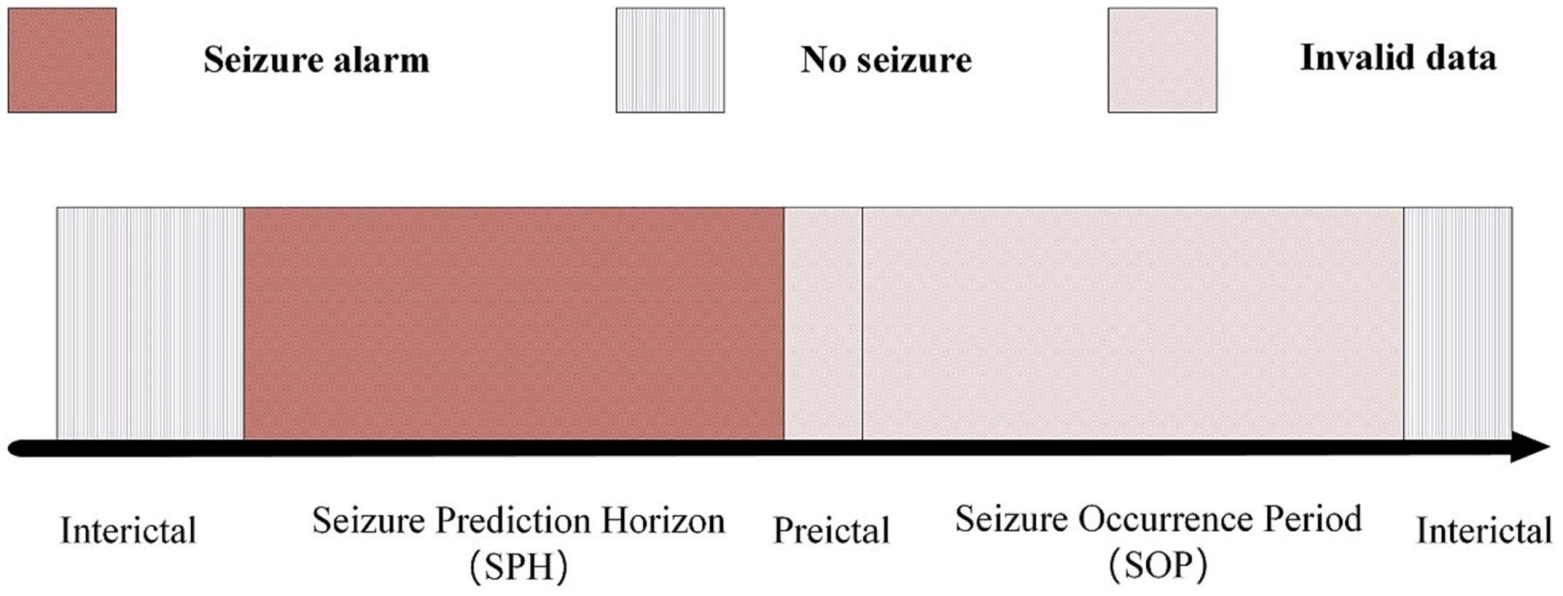
Epilepsy different stages state diagram.

Truong et al. (2018) established the time interval of SPH from 5 min before the actual seizure occurrence. Within this period, patients were provided only 5 min to prepare. To provide ample time for both the physician and the patient, we defines the SPH range as 15 min prior to the seizure up to 5 min before the seizure itself, as illustrated in Figure 4.

In present work, positive samples are retrieved during the 15 to 5 min interval leading up to seizure onset, as seizures are likely to occur during the following 15 min. Negative samples are segments without signs of imminent seizure within 15 min. The number of positive and negative samples of the dataset used in CHB-MIT was 11,300:11,400, which was generated because the time window used in this study was 6 s and each patient had a large number of EEG recordings, each recordings is at least two hours. To balance the positive and negative samples, the same 1:1 positive and negative samples were used for each patient.

### 3.1 Three-dimensional feature construction of EEG

**Figure 5:**
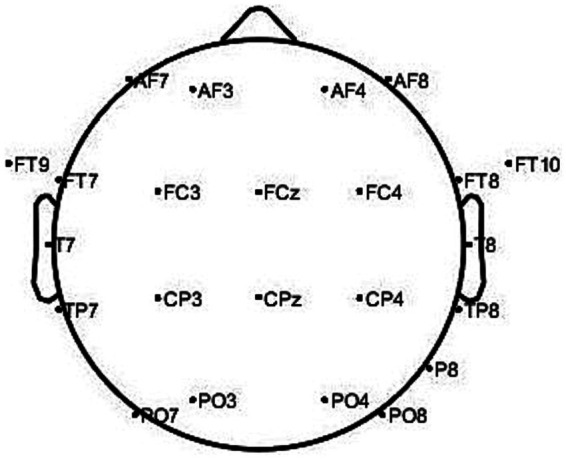
23 electrodes scalp localization map.

The CHB-MIT uses 23 electrodes for recording, which conforms to the positioning and naming of the international 10–20 system for EEG electrode placement standards. The dataset’s electrode names are as follows: AF7, AF3, AF4, AF8, FT9, FT7, FC3, FCz, FC4, FT8, FT10, T7, T8, TP7, CP3, CPz, CP4, TP8, P8, PO7, PO3, PO4, and PO8. Figure 5 shows a mapping of the actual spatial distribution of these scalp electrodes on the head.

**Figure 6:**
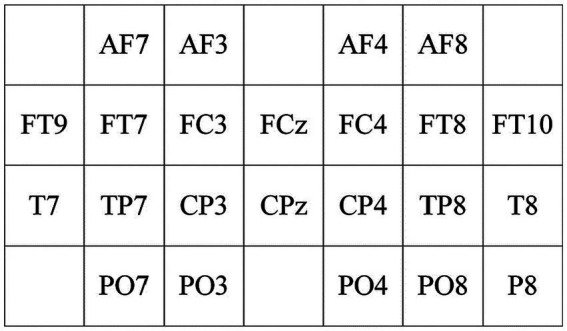
2D electrode channel positioning matrix diagram.

To extract spatial features between scalp electrodes, a spatial feature matrix with a 4 × 7 two-dimensional electrode channel is designed based on Figure 5, as illustrated in Figure 6. From Figure 6, the relative spatial relationships between different electrodes can be clearly understood.

**Figure 7:**
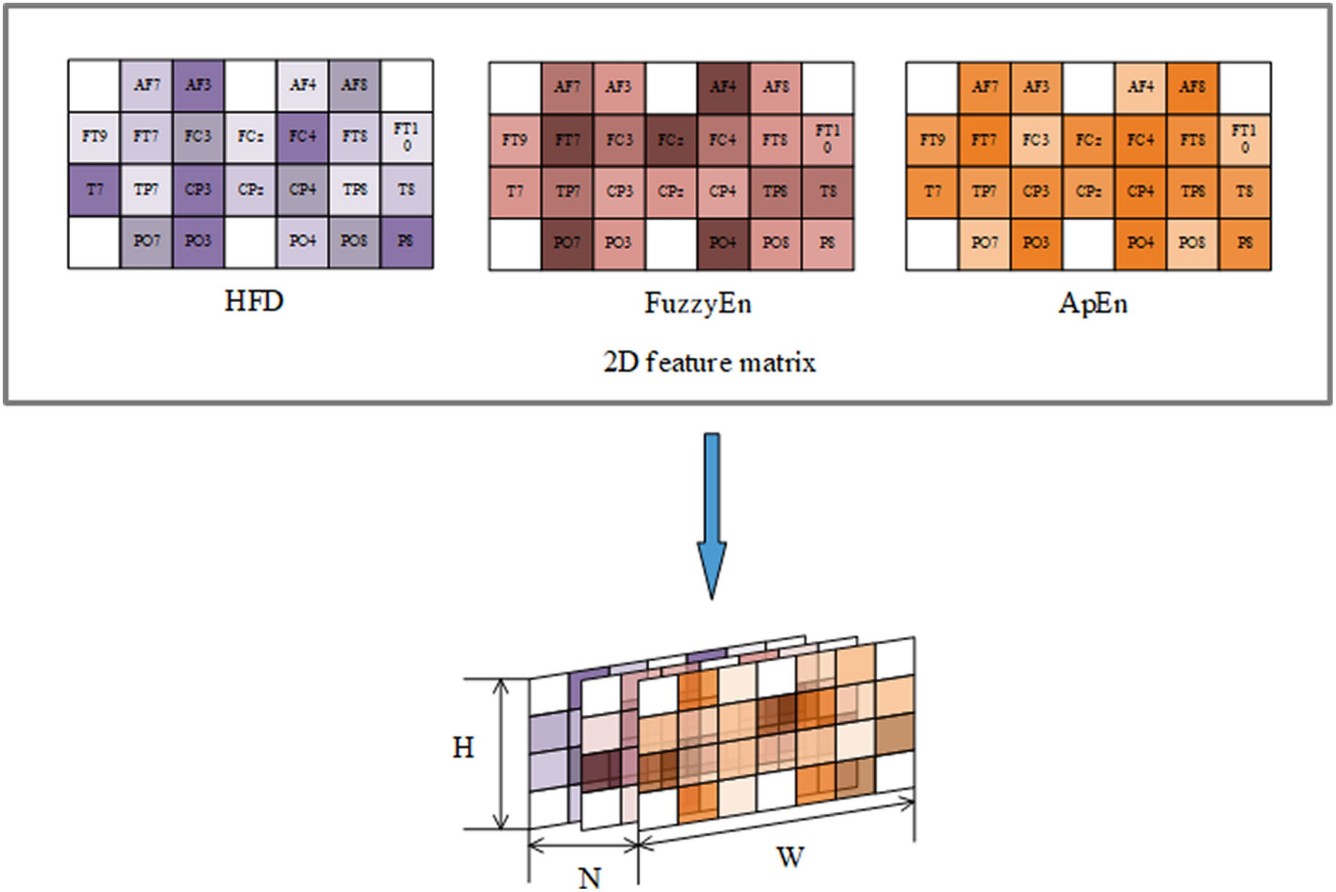
3D feature map.

To represent the EEG signals from a multi-feature perspective, the selected features were combined into a feature set: By arranging the 2D matrices of each feature after StandardScaler transformation, can obtain a 3D feature input composed of three features as illustrated in Figure 7. H represents the height of the matrix set to 4, W represents the width of the matrix set to 7, and N represents the number of selected important features, which is 3 in this case.

### 3.2 Pseudo-3DCNN structure learning

The proposed work utilizes a pseudo-3D CNN merged with a bidirectional ConvLSTM3D as the primary algorithm. By using 3D neural networks, the algorithm can preserve the electrode space information and information from multiple features. Assuming a conventional 3D convolutional kernel size is k∗k∗b , where k is the spatial dimension of the filter and b is the feature dimension of the filter, 3D convolution is computationally expensive and memory-intensive when learning features. In order to solve this problem, we can understand a 3D convolutional filter with a size of k∗k∗b as a k∗k∗1 convolutional filter for 2DCNN and a 1∗1∗b convolutional filter for 1DCNN. The k∗k∗1 convolution filter is used to obtain spatial information, while the 1∗1∗b convolution filter is used to obtain information about nonlinear characteristics. This method is called pseudo-3D (Qiu et al., 2017). In this study, we used pseudo-3D to extract EEG information from multiple dimensions, including spatial, nonlinear, and temporal. This not only reduces the computation and complexity of 3D convolution, but also realizes a more sensible feature extraction process. In order to extract the feature information of each nonlinear feature, each feature uses a convolution kernel with the size of k∗k∗1 . Then, a convolution kernel of the size 1∗1∗b is used to extract the information between these features. Pseudo 3D networks can adopt different convolution kernel sizes, stride sizes, and padding methods in both temporal and spatial dimensions to meet different needs.

### 3.3 P3DCNN-BiConvLstm3D-Attention3D model

It is equally important to extract temporal information for EEG while extracting spatial and feature information. Traditional BiLSTM is usually used to capture temporal correlations when processing temporal data, but it cannot effectively preserve spatial information features in the data. In contrast, Bi-ConvLSTM3D can simultaneously extract spatial relationships and temporal correlations from the data. This type of network is particularly suitable for the data in this study and can better handle 3D type data. Therefore, this study used Bi-ConvLSTM3D.

The purpose of this study was to determine whether the EEG signals belonged to SPH segments. Segments from 15 min before the seizure to 5 min before the seizure were designated as positive samples, and the remaining segments were designated as negative samples. For continuously recorded EEG signals, the data need to be segmented. In this paper, the EEG data were segmented into 6-s segments using a non-overlapping sliding window method.

**Figure 8:**
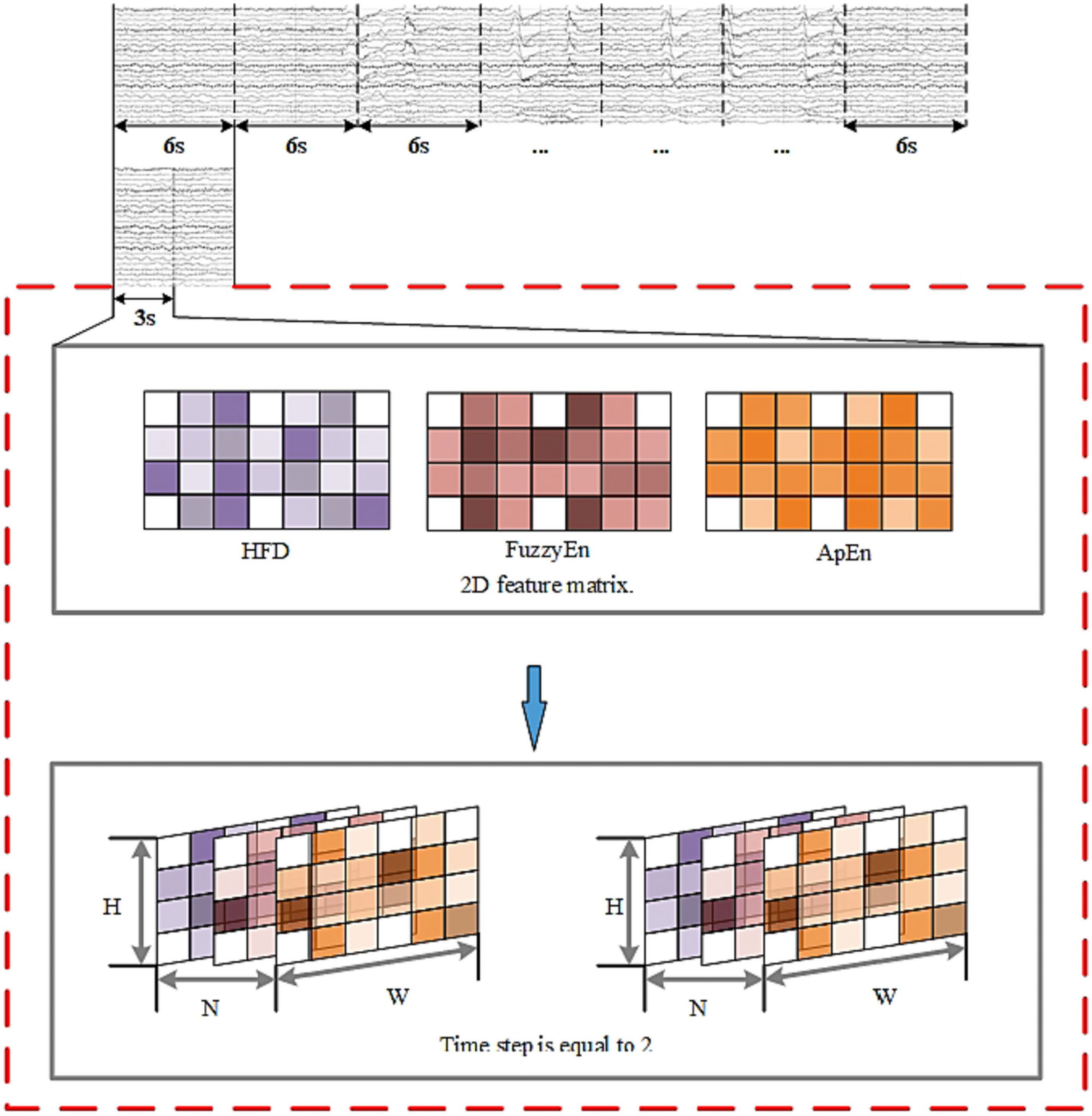
EEG segmentation and 3D feature construction.

**Figure 9:**
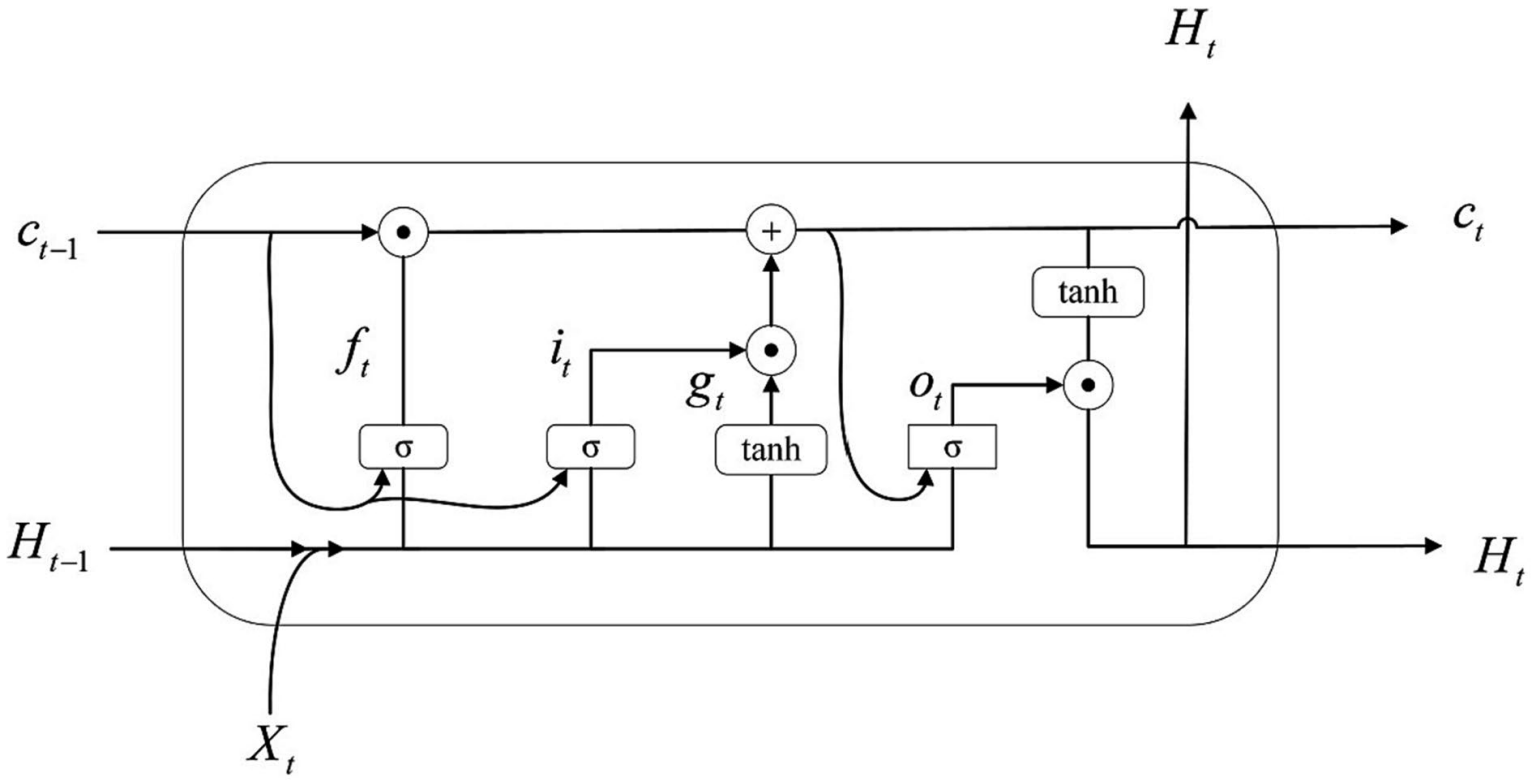
the structure of ConvLSTM3D cells.

**Figure 10:**
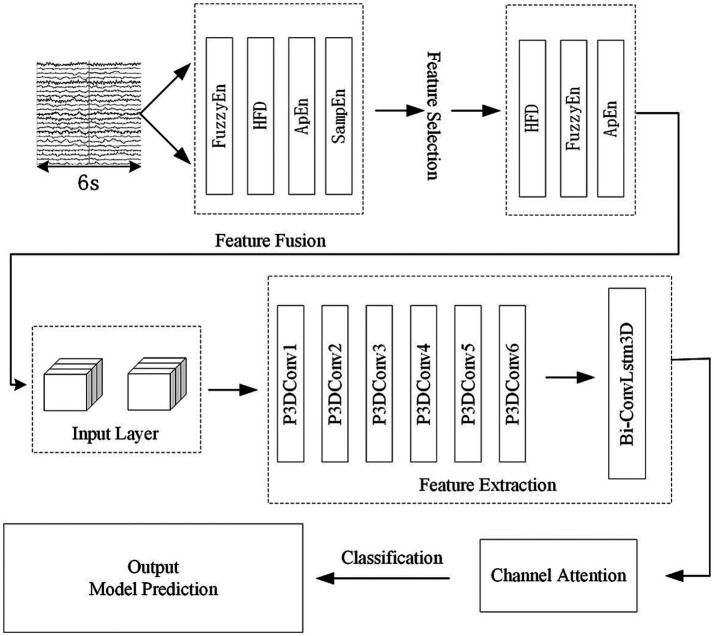
Overall architecture of the model.

The algorithm flow is illustrated in Figures 8–10 and the steps of alogrithm as Eqs. (19–24).

ConvLSTM3D is defined as Li et al. (2022):


(19) $$i_{t}=\sigma \left(W_{\mathrm{Xi}}*X_{t}+W_{Hi}*H_{t-1}+W_{ci}⊙c_{t-1}+b_{i}\right)$$

(20) $$f_{t}=\sigma \left(W_{Xf}*X_{t}+W_{Hf}*H_{t-1}+W_{cf}⊙c_{t-1}+b_{f}\right)$$

(21) $$g_{t}=\tanh\left(W_{X_{c}}*X_{t}+W_{H_{c}}*H_{t-1}+b_{c}\right)$$

(22) $$c_{t}=f_{t}⊙c_{t-1}+i_{t}⊙g_{t}$$

(23) $$o_{t}=\sigma \left(W_{Xo}*X_{t}+W_{Ho}*H_{t-1}+W_{co}⊙c_{t}+b_{0}\right)$$

(24) $$H_{t}=o_{t}⊙\tanh\left(c_{t}\right)$$

Where $X_{t}$ represents the three-dimensional characteristics of each time window, and the $H_{t}$ represents the hidden state. σ represents the Sigmoid function, and i,f,o correspond to the input gate, forget gate, cell gate (Li et al., 2022). The weights and biases indexed by X,H,i,f,o,c are learned through backpropagation. The symbol ⊙ represents matrix multiplication. The symbol ∗ represents the convolution operation. Figure 9 illustrates the details of this implementation.

The whole model includes the construction and input of three-dimensional EEG, spatial and nonlinear feature extraction, temporal feature extraction, channel attention mechanism and classifier. The training process is as follows: Use the features selected by mRMR to construct 3D features. The time-step is set to 2 and each step lasts for 3 s. The time distribution layer is used to wrap the pseudo-3DCNN, which can independently apply the layers or networks of the neural network to each time step of the sequence. We use Keras’ TimeDistributed layer to achieve this function. Then, the data is extracted through a temporal feature extraction layer to obtain temporal correlations. Finally, useful channels are enhanced by the 3D channel attention mechanism, and then it is sent to the full connection layer to determine the category of data segments. Table 1 illustrates the architecture of the pseudo-3D CNN layers; the temporal feature extraction layer takes two 3D blocks from the output of the convolutional layers as input, and the Bi-ConvLSTM3D layer captures temporal features and spatial information while preserving spatial information. Table 2 illustrates the architecture of RNN.

**Figure 11:**
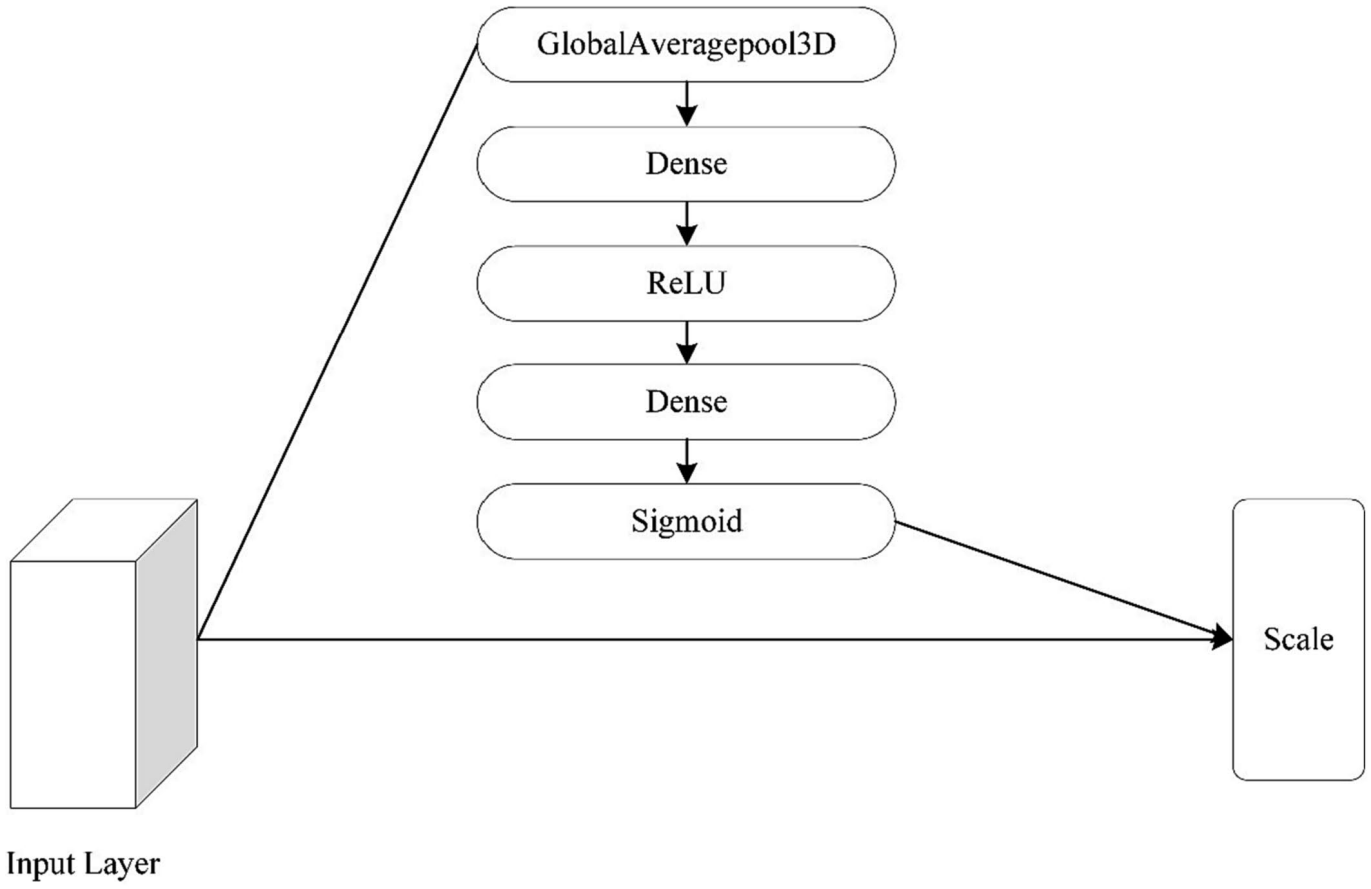
The architecture of convolutional block attention module.

The attention mechanism was also changed to a 3D type of attention mechanism, as the focus of this paper is on the 3D module. The specific operation of the channel attention mechanism is as follows: Squeeze-Excitation-Scale (Hu et al., 2018). The squeeze is the use of global average pooling to compress the three-dimensional features of each channel into a real number. The excitation is to generate a weight value for each channel, use two fully connected layers and an activation layer to construct the correlation between channels, and output the same number of weights and channels. The Scale is the process of weighting the active weights to each channel. Figure 11 shows the network structure of the channel attention mechanism.

In the P3DCNN model, batch normalization is performed after every two pseudo-3DCNN layers. In this paper, the number of parameters for pseudo-3DCNN is 364,720, the number of parameters for Bi-ConvLSTM3D is 1,050,624, and the number of parameters for Attention3D is 66,112.

### 3.4 Evaluation criteria

In present work, the performance of the classification model is evaluated using the cross-validation method, the main idea of which is as follows: for all the data, divide it into k subsets of unrelated samples, i.e., $D=D_{1}\cup D_{2}\cup \ldots \cup D_{k_{i}},D_{i}\cup D_{j}=\emptyset \left(i\neq j\right)$ , and then each time select a subset of samples to be used for testing, and the rest of the data are all used for training, and after k times of training in this way, average the k results and calculate the final result. In this paper sets k to 5. And independently conduct five 5-fold cross-validation to obtain the average ACC and STD to evaluate the performance of the model.

Choosing appropriate evaluation criteria is beneficial for enhancing the credibility of model performance. In addition, to evaluate the effectiveness of the epilepsy prediction models in this study, accuracy (Acc), sensitivity, precision and specificity were used as evaluation metrics, these metrics as Eqs. (25–28).


(25) $$Acc=\frac{TP+TN}{TP+TN+FP+FN}$$

(26) $$\mathrm{Sensitivity}=\frac{TP}{TP+FN}$$

(27) $$\mathrm{Precision}=\frac{TP}{TP+FP}$$

(28) $$\mathrm{Specificity}=\frac{TN}{FP+TN}$$

Where TP, TN, FP, FN represents true positives, true negatives, false positives, and false negatives.

## 4 Results and discussion

The proposed model in this paper is compared with other baseline methods on the CHB-MIT dataset. The details and parameters of these methods are as follows.

SVM: Support Vector Machine is a supervised machine learning model for general linear classification. It is widely used for both classification and regression tasks. SVM maps the feature vectors of instances into points in space and then separates these points with a hyperplane for classification. SVM is suitable for small to medium-sized datasets, as well as nonlinear and high-dimensional classification problems. Since this study involves a three-dimensional feature structure, SVM was chosen as one of the baseline methods. The kernel of SVM is set to ‘rbf’, and the decision_function_shape is set to “ovo”.

K-Nearest Neighbors (KNN): The basic idea of this algorithm is to compare the attribute features of the test dataset with the corresponding attribute features in the training dataset. In the training dataset, it finds the k nearest “neighbors” and determines the class of the test dataset sample based on the majority class among these k neighbors. In this comparative experiment, the value of n_neighors used for KNN is set to 5, and the metric is set to ‘minkowski’.

**Figure 12:**
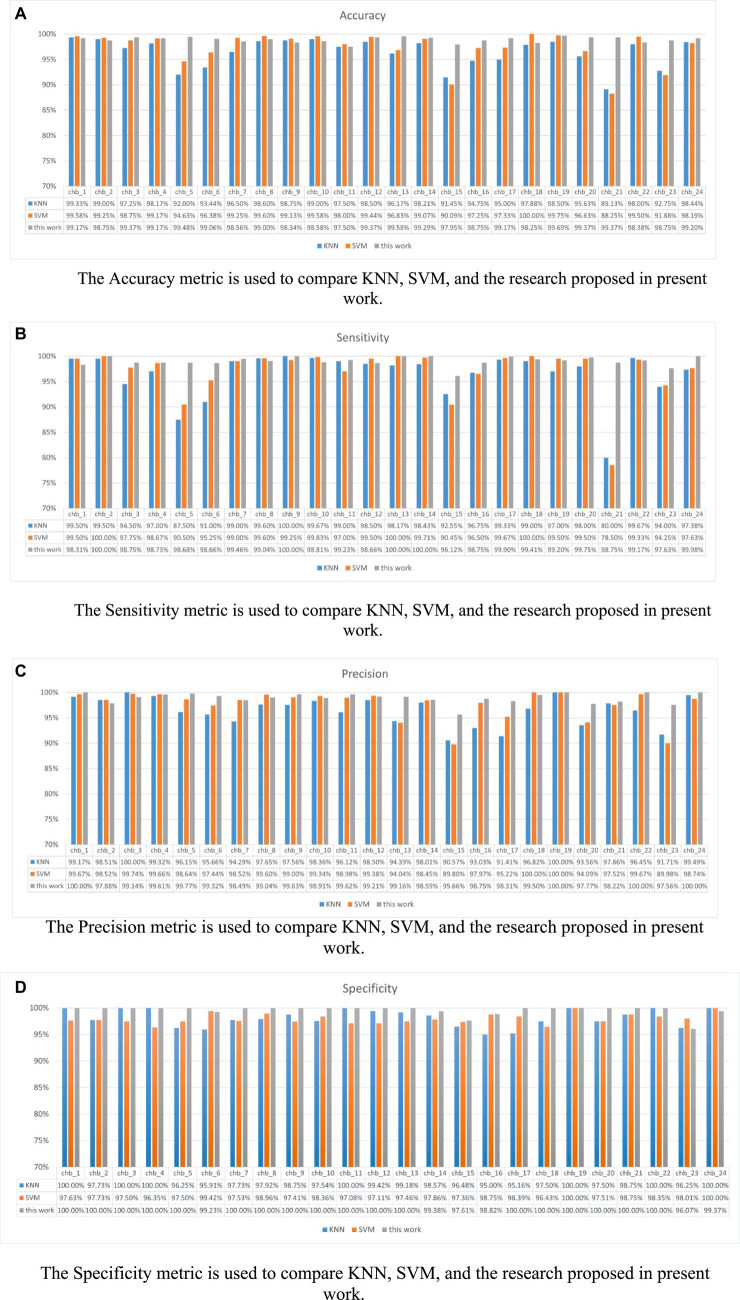
Shows the metrics for each subject under different models. **(A)** Accuracy **(B)** Sensitivity **(C)** Precision **(D)** Specificity. **(A)** The Accuracy metric is used to compare KNN, SVM, and the research proposed in present work. **(B)** The Sensitivity metric is used to compare KNN, SVM, and the research proposed in present work. **(C)** The Precision metric is used to compare KNN, SVM, and the research proposed in present work. **(D)** The Specificity metric is used to compare KNN, SVM, and the research proposed in present work.

Figure 12 shows the performance of KNN, SVM, and P3D-BiConvLstm3D-Attention3D on the CHB-MIT dataset. From Figure 12, it can be observed that the proposed model in this study demonstrates effectiveness and generalizability on the CHB-MIT dataset. The top two rows and the last row of Table 3 depict the average ACC and STD of these methods. We can observe that compared with the other baseline methods, our model achieves the highest ACC and the lowest STD. Our model has an accuracy of 98.13%, which is 1.72% and 0.74% higher than KNN and SVM, respectively.

**Table 3:** Performance ( $\bar{Acc}\pm$ Std(%)) of traditional machine learning models and the proposed model on the CHB-MIT dataset.

Method	Input feature	Window size	CHB-MIT $\bar{Acc}\pm$ Std(%)
KNN	1D vector	6 s	96.41 ± 2.81
SVM	1D vector	6 s	97.39 ± 3.17
The proposed work	4D mesh sequence	6 s	98.13 ± 0.00

Our model outperforms KNN and SVM in most patient metrics, with accuracy, sensitivity, specificity, and precision metrics of over 95% across all patients. Regarding the performance of the method proposed in this study on the dataset, the accuracy of this method is 98.13%, sensitivity is 98.03%, precision is 98.30% and specificity is 98.23%. Therefore, we have reason to believe that our proposed model is reliable. The experimental results show that our p3DCNN-BiConvLSTM3D-Attention3D model achieves better performance than traditional machine learning algorithms (SVM and KNN).

Table 4 presents a comparison of the results between this study and other research papers. Zhang et al. (2020) used Pearson correlation coefficients as features and then employed a convolutional neural network for prediction, achieving an accuracy of 89.98%, which is lower than the accuracy achieved in this study. This suggests that incorporating spatiotemporal features can indeed improve the accuracy of epilepsy prediction. Das et al. (2020) utilized a framework consisting of various feature extraction algorithms (lower threshold, target point selection, and current maxima), energy features, and pattern matching (segment and domain). The authors’ model proposal, power, homogeneity, maxima, energy, and physiological traits have been employed. The algorithm achieved an accuracy rate of 92.66%, a F1-score rate of 94.86%. Muhammad Usman et al. (2021) utilized a three-layer custom convolutional neural network in combination with handcrafted (temporal and spectral) features. The feature set was used to train an ensemble classifier, which integrated the outputs of SVM, CNN, and LSTM. On the CHB-MIT dataset, the average sensitivity rate achieved was 96.28%, the average specificity rate achieved was 95.65%. Muhammad Usman et al. (2020) utilized short-time Fourier transform (STFT) to extract frequency-domain and time-domain information from 30-s EEG windows. A neural network was utilized to classify segments between pre-seizure and interictal periods. On the CHB-MIT dataset, the sensitivity rate achieved was 92.7%. the specificity rate achieved was 90.8%. Singh and Malhotra (2022) proposed a two-layer LSTM network model that utilized the spectral power and average spectral magnitude features of α, β, γ, θ, δ bands from a 23-channel EEG spectrum. The model achieved an average accuracy rate of 98.14%, an average sensitivity rate of 98.51%, an average specificity rate of 97.78%. Zhang et al. (2021) combined with multidimensional sample entropy and Bi-LSTM, the seizure prediction accuracy was 80.09% and the FPR was 0.26/h. Prathaban and Balasubramanian (2021) reconstructed the EEG with sparsity and converted it into a two-dimensional image. Then, to account for the relationship between channels, the two-dimensional image was converted into a three-dimensional image of time, signal value, and channel representation, and a three-dimensional optimized convolutional neural network was used to predict seizures with an accuracy of 0.98%, sensitivity of 0.99%, and False Prediction Rate (FPR) of 0.07 FP/h.

**Table 4:** Comparison of the performance of existing epilepsy prediction methods.

Reference	Methods	Results
Zhang et al. (2020)	Pearson correlation coefficient, CNN	Accuracy: 89.98%
Das et al. (2020)	Statistical features, pattern matching	Accuracy: 92.66%,
Muhammad Usman et al. (2021)	Three-layer CNN, handcrafted Features, SVM, CNN, and LSTM	Sensitivity: 96.28%, Specificity: 95.65%
Muhammad Usman et al. (2020)	STFT, CNN	Sensitivity: 92.7%, Specificity: 90.8%
Singh and Malhotra (2022)	Two-layer LSTM, spectral power, spectral magnitude features	Accuracy: 98.14% Sensitivity: 98.51%, Specificity: 97.78%
Zhang et al. (2021)	Multidimensional SampEn, Bi-LSTM	Accuracy: 80.09%
Prathaban and Balasubramanian (2021)	Dynamic learning framework, sparsity based EEG Reconstruction, three-dimensional Optimized CNN	Accuracy: 98%, Sensitivity: 99%
This proposed work	Nonlinear feature, Select by mRMR, Pseudo-3D, Bi-ConvLSTM3D	Accuracy: 98.13%, Precision: 98.30%, Sensitivity: 98.03%, Specificity: 98.23%

## 5 Conclusion

We have proposed a seizure prediction algorithm that combines multiple feature selections and pseudo-3D neural networks. This method extracts multiple features and combines them to form unique 3D features. It uses multi-layer pseudo-3D convolutional neural networks, BiConvLSTM3D, and 3D channel attention mechanisms for automatic detection. The accuracy of this method is 98.13%, sensitivity is 98.03%, precision is 98.30% and specificity is 98.23%. The method outperforms most advanced similar methods with high sensitivity and a prediction time of 15 min in advance. Compared to other methods, our results indicate that our model has similar or better predictive accuracy, sensitivity, accuracy and specificity, which further validate the effectiveness of our method. However, there is still room for improvement in many areas. Grid search can be applied to the model to systematically search for the optimal combination of hyperparameters for optimal performance. In this study, all scalp electrode channels were used, and future research will further investigate the optimization of multi-channel epilepsy. In three-dimensional neural networks, parameters can be reduced, computational efficiency can be improved, and the maximum information can be expressed with the least number of electrodes. In addition, the gender and age distribution of patients will be incorporated into the three-dimensional features to further investigate the relationship between gender, age, and epilepsy prediction.

## Data availability statement

The original contributions presented in the study are included in the article/supplementary material, further inquiries can be directed to the corresponding authors.

## Author contributions

XL, ZYL, and LSZ designed the work and wrote this original manuscript. LSZ and XCL contributed to the review and editing. CYL and HHK contributed to optimize of problem definition ZYL was mainly responsible for this project. All authors contributed to the article and approved the submitted version.
